# Diabetes mellitus is associated with poor self-rated health in olders compared to other chronic conditions

**DOI:** 10.1186/1758-5996-7-S1-A203

**Published:** 2015-11-11

**Authors:** Valéria Pagotto, Erika Aparecida da Silveira

**Affiliations:** 1 Universidade Federal de Goiás, Goiânia, Brazil

## Background

Self-rated health is one of the most widely used indicators to estimate healthn conditions of a population. There is evidence of its reliability and its potential in predicting mortality, and functional decline. However, little is known of which chronic diseases that most affect the self-perception of health of the elderly.

## Objectives

To estimate the prevalence of self-rated poor health and their association with chronic conditions in elderly.

## Materials and methods

This is a cross-sectional study, with older adults (>60 anos), users of the Unified System of Health. The sample was held in multiple stages, from the Health Districts of Goiânia-GO. Data were collected from December 2008 to March 2009. Self-rated health was dichotomized in self-rated good health (very good/good/fair) and bad (poor/very poor). Chronic diseases were identified through the responses to the question: “Which diseases the doctor has said that you have?” The relationship between self-rated poor health and the chronic conditions were explored through the prevalence ratio (PR). Multivariate analysis was performed using Poisson regression with hierarchical analysis. Were included in this analysis the variables with value p <0.20. The tests were performed in STATA 12.0.

## Results

From 403 elderly, 66% were female, 29.8% aged 65-69 and 28.8% with less than 1 year of study. Self-rated poor health was reported by 27.5% of the elderly, with higher prevalence among women (29.7%) and aged 60-64 yrs. (29.1%). Poor self-rated health was associated with: 3 or more morbidities (RP=1.98, 95%CI 1.36-2.90), have diabetes (RP=1.57, 95%CI 1.05-2.34), musculoskeletal diseases (RP=1.64; 95% CI 1.11-2.45), have been hospitalized in the past year (RP=1.68; 95%CI 1.14-2.49) and polypharmacy (RP=1.70; 95%CI 1.07–2.68). In multivariate analysis, the highest RP were: diabetes (RP=.52, 95% CI 1.02-2.27) and musculoskeletal diseases (RP=1.84, 95%CI 1.26 to 2.68).

## Conclusions

The result shows that Diabetes Mellitus and musculoskeletal diseases are associated with poor self-rated health. Both conditions can lead to functional limitations, disability and require a variety of health care, in particular Diabetes Mellitus. These factors can generate a sense of bad health conditions, and take the elderly mean a bad health. Therefore, this is an indicator that can be evaluated in older people with diabetes, and musculoskeletal diseases either in clinical practice, as in the management of health services.

